# Micro-CT Comparative Assessment of Quartz Fiber Hollow and Solid Posts for the Restoration of Endodontically Treated Teeth

**DOI:** 10.3390/jcm14217725

**Published:** 2025-10-30

**Authors:** Luca Mirabelli, Edoardo Bianco, Fabio Sebeto, Claudio Luigi Citterio, Alberto Pellegatta, Marcello Maddalone

**Affiliations:** Dental Clinic, Department of Medicine and Surgery, University of Milano Bicocca, 20900 Monza, Italy; luca.mirabelli87@gmail.com (L.M.); f.sebeto@campus.unimib.it (F.S.); claudio.citterio@unimib.it (C.L.C.); alberto.pellegatta@unimib.it (A.P.); marcello.maddalone@unimib.it (M.M.)

**Keywords:** endodontics, fiber post, hollow post, Micro-CT, restoration, quartz post

## Abstract

**Background:** Hollow posts have been introduced in clinical practice, providing the possibility of injecting luting resin directly into the post. The aim of our study was to compare quartz fiber hollow posts with solid posts. **Methods:** In total, 20 human teeth with straight single root canals were utilized for this study, divided into two groups with 10 elements each, one restored with radiopaque quartz fiber hollow posts and the other with radiopaque quartz fiber solid posts. In total, two micro-CT (micro-computerized tomography) analyses allowed us to evaluate both the presence of air voids in the luting resin and the different capacities of posts to penetrate until full depth into the post space. **Results:** The authors observed that hollow quartz fiber posts create a smaller volume of air voids in the luting resin (*p* < 0.01) and better fit (*p* < 0.05) the post space compared to the solid posts. **Conclusions:** Hollow posts can promote retention. Future studies with larger samples are encouraged to confirm these findings and provide possible better long-term results for post-endodontic reconstructions in vivo.

## 1. Introduction

Root-filled teeth usually present a higher risk of loss than teeth with vital pulps due to vertical and lateral masticatory forces and the subtraction of coronal and radicular tooth structure; in fact, over 70% of root fractures occur in endodontically treated teeth [1,2,3,4].

In the case of fracture, teeth could be forced to be extracted due to the poor prognosis of reconstructions or even the complete impossibility of recovery, resulting in the need for a prosthesis or surgical procedures such as autotransplantation or implantology [5,6,7].

In order to improve the long-term prognosis of endodontically treated teeth, posts of various shapes and sizes have been widely used in dentistry to act as retention devices for tooth reconstruction [8,9], providing retention for the core material after the loss of a large amount of dental structure and creating a biomechanically stable and homogenous root canal–post–resin monoblock, improving restoration survival and fracture resistance [10].

In the past, the principal type of post used was a metal post (cast or prefabricated), which showed a high elastic modulus: in fact, stainless steel and titanium were considered strong and clinically effective [11]. Traditional metallic posts have been gradually replaced with fiber posts (glass, quartz, and carbon-reinforced composites) due to several advantages, such as a better elastic modulus, enhanced biocompatibility, better esthetics, and higher corrosion resistance with a decreased number of irreparable root fractures [4,12].

Many authors have reported that metal posts seem to induce root fractures more frequently than fiber posts [4,11], likely due to the significant discrepancy between the elastic modulus of metal (approximately 200 GPa) and dentin (approximately 42 GPa) [10,11]. In contrast, the elastic modulus of fiber posts (25–57 GPa) is much closer to that of dentin [13]. Therefore, it’s clear that a device with physical and mechanical properties as similar as possible to dentin is required.

The characteristics of fiber posts, particularly the length and diameter of the posts and the thickness of the adhesive cements, and the clinical procedures adopted, strongly influence the performance of the restorations. The polymerization of the luting resin leads to centripetal contraction, which is one of the primary causes of air void formation [14]. The polymerization process is of great importance, as the resin compounds must be activated to achieve the conversion of monomers into polymeric chains; variations in polymerization levels significantly influence the bond of resin luting agents to root dentin. To overcome this limitation, dual-cured luting resins have been developed; the polymerization process is initiated by photoactivation and then continues through the action of a self-curing catalyst, thus combining the advantages of both photo- and self-cured luting agents [15].

Recently, the properties of hollow posts have been investigated. The ability to inject composite cement directly through the post makes the positioning and luting procedures simultaneous and the technical steps more predictable. Furthermore, the formation of a solid composite core within the hollow portion of the post creates a more cohesive post-resin system by increasing the internal contact surface area. The use of a hollow post reflects the engineering principle of a sandwich structure: when stressed by mechanical forces, tension is generated in the bottom skin and compression in the top skin, while the central core experiences minimal solicitation [16].

The aim of this study was to evaluate the presence of air voids in the luting resin when using two different kinds of fiber posts, a hollow and a solid one, with the use of micro-CT (micro-computed tomography). This technology allows for a 3D quantitative and qualitative evaluation of the prepared root canals in order to determine if there are differences in clinical performance between these two types of fiber posts.

Based on the aforementioned challenges and the potential advantages of hollow posts, this study aimed to investigate specific differences using micro-CT analysis. The null hypotheses were formulated as follows: (1) The volume of air voids in the luting resin will not differ significantly between restorations using hollow quartz fiber posts and those using solid quartz fiber posts. (2) There will be no significant difference in the penetration depth achieved by hollow versus solid quartz fiber posts within the prepared post space.

## 2. Materials and Methods

**Figure 1:**
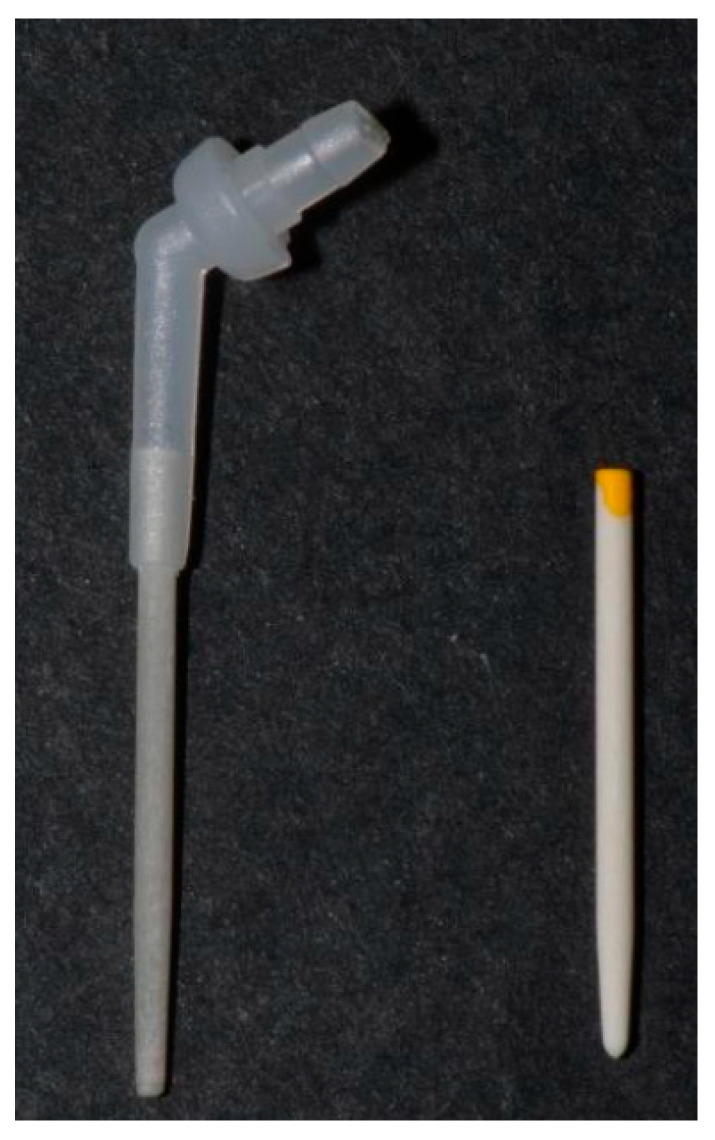
Hollow post (TECHOLE size S, Isasan, Rovello Porro, Italy) and solid post (TECH21XOP Ø12, Isasan, Rovello Porro, Italy).

To evaluate the formation of air voids within the luting resin, two different posts were studied: a solid post (TECH21XOP Ø12, Isasan, Rovello Porro, Italy) and a hollow post (TECHOLE size S, Isasan, Rovello Porro, Italy), both luted with the same luting resin (NEW TECHCORE A, Isasan, Rovello Porro, Italy) (Figure 1).

A total of 20 human single-rooted teeth with straight root canals were collected and stored for 48 h in a 0.9% NaCl physiological saline solution. Inclusion criteria were as follows: completely formed teeth with closed apices; single roots; and the absence of root fractures, caries, or other pathologies that could interfere with the study outcomes. All teeth were sectioned perpendicular to their long axis with a diamond disk to achieve a final length of less than 20 mm for each sample.

To ensure procedural consistency and eliminate inter-operator variability, all clinical procedures were performed by a single experienced operator. Endodontic treatment was performed using Ni-Ti rotary instruments (MTWO, Sweden&Martina S.p.A., Vimercate, Italy) up to a final size of 25 with a 0.06 taper. The chemo-mechanical debridement protocol included irrigation with 5% sodium hypochlorite (Niclor 5, OGNA, Milan, Italy), followed by a final rinse with 17% ethylenediaminetetraacetic acid (EDTA, OGNA, Milan, Italy) to remove the smear layer.

The canals were obturated using the warm vertical compaction technique (continuous wave of condensation). After fitting a non-standardized gutta-percha master cone, Pulp Canal Sealer™ (Kerr Dental, Brea, CA, USA), a zinc oxide-eugenol-based cement, was applied. The apical third was sealed by compacting the gutta-percha with an electrically heated plugger to within 4–5 mm of the working length. The remainder of the canal was then backfilled with injectable, thermoplasticized gutta-percha.

Following obturation, a post space was created using dedicated burs, ensuring that 4–5 mm of gutta-percha remained as an apical seal. Gates-Glidden drills (No. 4 and 5; Dentsply Maillefer, Brea, CA, USA) were subsequently used to remove any remaining gutta-percha debris from the canal walls.

**Figure 2:**
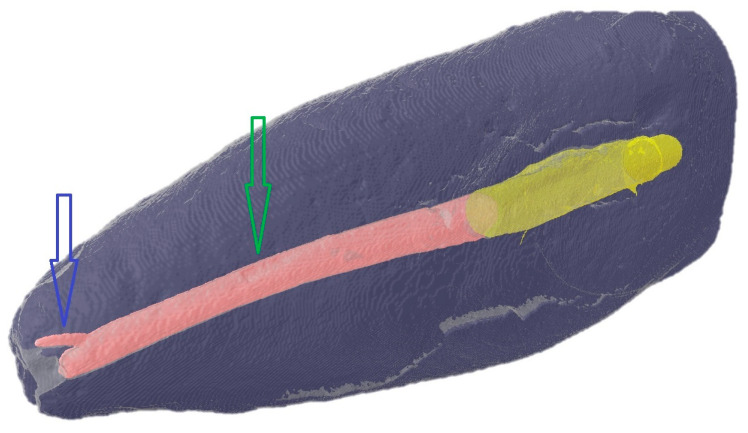
Correct apical seal (blue arrow) and canalar seal (green arrow) on a CT-Scan. Yellow: coronal third of the root canal; pink: apical and middle third of the root canal.

All specimens underwent an initial micro-CT scan at this stage, so that images of the elements before and after post-luting could be superimposed using DataViewer™ software (Adelaide, Australia, Version DV16) (Figure 2).

Subsequently, all post spaces were rinsed with 17% EDTA for 45 s, dried with paper points, and then etched for 30 s with 37% phosphoric acid gel. After rinsing for 20 s and gentle air-drying, a total-etch dual-cure adhesive resin (New TECHBOND DC, Isasan, Rovello Porro, Italy) was applied with a microbrush, followed by air-thinning to remove excesses.

The specimens were randomly divided into two groups of 10 teeth each, and the posts were luted according to the manufacturer’s recommendations:

o Elements belonging to Group 1 (HP) were restored with radiopaque quartz fiber hollow posts (TECHOLE size S, Isasan™, Rovello Porro, Italy). The luting technique involved seating the hollow post to the full depth of the post space. With these posts, it is not necessary to first fill the canal with luting resin; instead, the procedure is performed in a single step where the post simultaneously acts as a guide for the luting resin. The micro-hybrid dual-curing luting composite (NEW TECHCORE A, Isasan™, Rovello Porro, Italy) was injected through the post itself using an automixing syringe equipped with a dedicated adapter, followed by light-curing for 40 s.
o Elements belonging to Group 2 (SP) were restored with radiopaque quartz fiber solid posts (TECH21XOP Ø12, Isasan™, Rovello Porro, Italy). The micro-hybrid dual-curing flowable luting composite (NEW TECHCORE A, Isasan™, Rovello Porro, Italy) was first injected into the post space using the manufacturer-provided automixing syringe. The solid post was then inserted to its full depth with sustained finger pressure for 30 s, followed by light-curing for 40 s.

### 2.1. Micro-CT Analysis

Following the restorative procedures, all specimens underwent a second micro-CT scan. The pre- and post-restoration scans were superimposed using DataViewer™ software (Adelaide, Australia, Version DV16). The resulting volumes were analyzed using CTAn software (Bruker CTAn Micro-CT Software, version 1.13, Cambridge, UK). The micro-CT equipment used for the analysis was a SkyScan 1176 (Bruker, Cambridge, UK), with the technical specifications detailed in Table 1.

The total volume of air voids was calculated by subtracting the volume of the filled portions (post and cement) from the total volume of the prepared post space.

**Figure 3:**
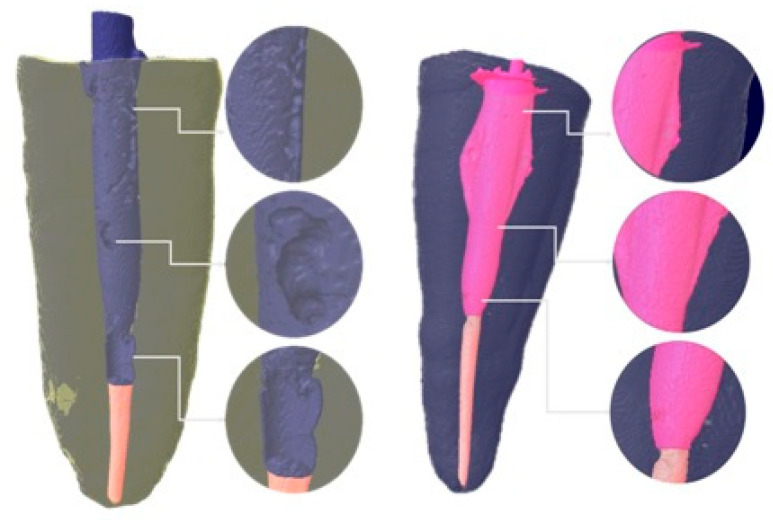
Different void formations in the post space with solid posts on the left and hollow posts on the right.

**Figure 4:**
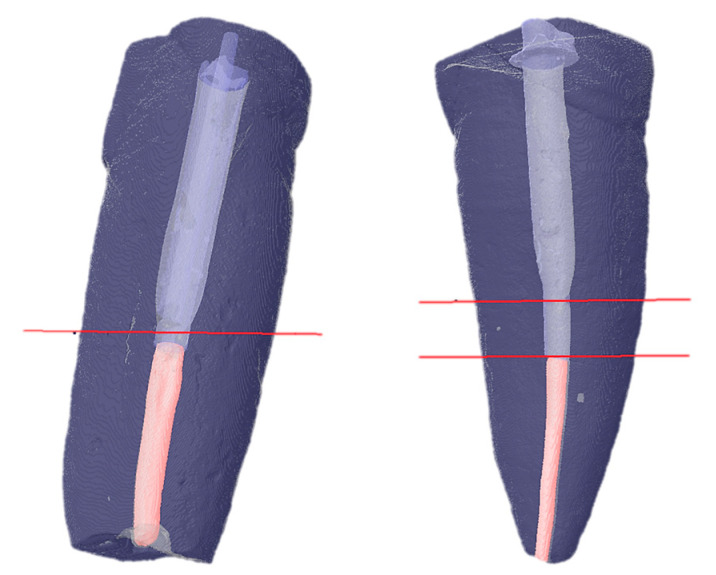
Differing penetration of posts: on the left, a hollow post (red line indicates the portion of post space not reached by the post) and on the right, a solid post (double red line indicates the portion of post space not reached by the post).

Two comparative assessments were performed:

1. Hp1: Analysis of the volume of air voids within the luting cement (Figure 3).
2. Hp2: Analysis of the difference between the HP and SP groups in their ability to reach the most apical portion of the post space (Figure 4).

### 2.2. Statistical Analysis

Statistical analysis was performed using GraphPad Prism 7.0. A total of 20 human teeth were utilized for this study, divided into 2 groups of 10 teeth each. Although a formal a priori sample size calculation was not conducted, a post hoc power analysis was performed. To assess the distribution of the data for both hypotheses, the D’Agostino-Pearson omnibus test and the Shapiro–Wilk test were used. As both tests indicated that the data were not normally distributed, non-parametric statistical tests were employed. Specifically, the Mann–Whitney U-test for unpaired samples was used to evaluate the differences between the HP and SP groups for both air void formation (Hp1) and penetration depth (Hp2).

Based on the observed data for air voids (hollow posts mean: 0.0440, SD: 0.0274; solid posts mean: 0.1734, SD: 0.0832), the power analysis revealed a large Cohen’s d effect size of 2.09. With an assumed statistical power of 80% and a significance level of 0.05, the estimated minimum required sample size per group was 4, confirming that the study was adequately powered.

During the preparation of this manuscript, the authors used Claude AI [Claude 3.7 Sonnet version] for the purpose of English grammar correction.

## 3. Results

The data collected for the two groups (*n* = 10 each) were analyzed using GraphPad Prism 7.0 software. Normality testing via the D’Agostino–Pearson and Shapiro–Wilk tests confirmed that the data were not normally distributed.

The results regarding the presence of air voids in the luting resin (Hp1) are reported in Table 2.

The results regarding the different capacities of posts to reach the full depth of the post space (Hp2) are reported in Table 3.

**Figure 5:**
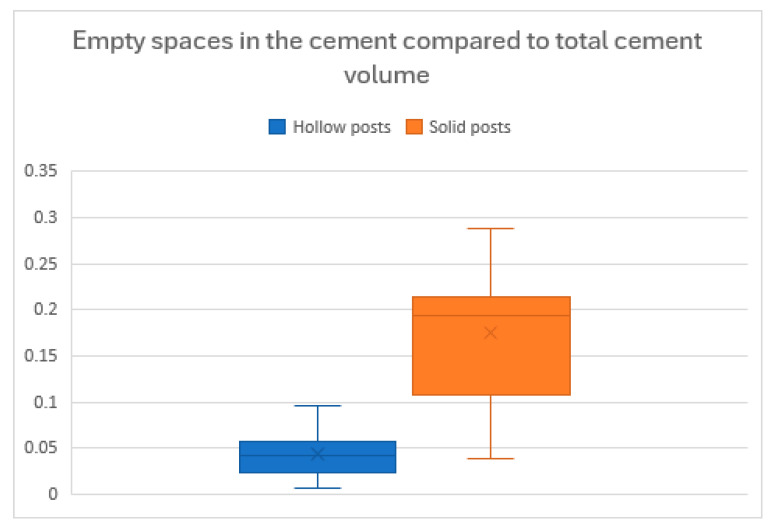
Empty spaces in the luting resin compared to total luting resin volume: difference between hollow and solid posts (box and whiskers plot with mean and SD; median and quartiles).

Since the data for Hp1 and Hp2 did not show a normal distribution, they were analyzed using non-parametric tests:

- Hp1: The Mann–Whitney U-test showed a statistically significant difference (*p* = 0.0007) between the HP and SP groups regarding the formation of air voids in the luting resin, with hollow posts yielding a better adhesive layer (Table 4). The null hypothesis is therefore rejected.
- Hp2: The Mann–Whitney U-test showed a statistically significant difference (*p* = 0.0433) between the HP and SP groups in their ability to reach the most apical portion of the post space (Table 5). This null hypothesis is also rejected (Figure 5).

## 4. Discussion

Posts placed in root canals of severely decayed teeth have been universally recognised to promote stable crown reconstruction [17]. Different properties, like shape, length, construction material of posts, luting resin used for post fixation, and reconstruction with or without full cuspid coverage, were investigated over time to achieve the best performance for placed posts [4,17].

Various authors affirm that restorations with composite or composite with fiber posts may be a better treatment modality for non-vital teeth compared to metal and/or ceramic posts: in fact, in the event of failure, the likelihood of deep tooth fractures is lower with composite or composite with fiber posts. A critical challenge to solve remains the adhesion into the root canal because of unfavorable ovoid or non-regular canal configurations, and even the dentin microstructure in the deepest part of the canal could be responsible for detachments over time [4,13].

Nevertheless, Figueiredo et al., in a meta-analysis of the literature regarding the incidence of root fractures, stated that there were no significant differences between metal and fiber posts [18].

In our study, we evaluated the performance of a new generation of tubular posts with the possibility to inject composite luting material even in the inner part of the post system in order to better simulate the modulus of elasticity of the surrounding dentin.

Fiber post debonding is the most common clinical failure that can occur, often requiring urgent dental treatment [19], even if several luting techniques are available [20]. Adhesion over time between fiber post, root dentine, and adhesive luting resin is critical for long-term performance [21]. The etch-and-rinse adhesive system is an effective protocol for the adhesion, with resin tags and adhesive lateral branches formation in the dentin walls [22].

Iniba et al. and Generali et al. reported a significantly better push-out bond strength for hollow posts with respect to solid posts, confirming the favorable characteristics of this kind of post [16,23]. Our study’s results, generated via a micro-CT analysis, showed an average void percentage in the luting resin of approximately 4.4% for the hollow post (HP) group and 17.3% for the solid post (SP) group. These data are consistent with the observations of Inaba et al. (2013), who, using a similar methodology, found a five-fold lower void volume in hollow posts compared to solid ones, confirming the superiority of the internal luting technique [16].

Other parameters useful for evaluating reconstructive hollow posts have been discussed in the literature. Khoroushi et al. compared various luting techniques for solid fiber posts, reporting very low void percentages (1.1–3.5%) with techniques that allow better control for luting resin injection, showing how the luting technique drastically influences the outcome. Bovolato et al. reported better compression and cutting resistance of hollow posts, and Lo Giudice et al. also reported better fracture, flexural, and deflexural resistance [24,25,26].

Besides these studies targeting the mechanical properties of posts, there are only a few studies regarding bubbles or voids in the root canal–post–resin system. Voids are places where no adhesion occurs and, at the same time, are potential areas of fracture and initial separation phenomena, so a reduction in void formation is a requirement for better seal and adhesion. Iniba et al., with a micro CT scan of lower premolar specimens, detected a number of voids five times lower in hollow posts with respect to solid posts [16].

Another characteristic that has to be considered is that hollow posts have an anatomical shape that implies a thinner and more uniform luting resin layer in the coronal and middle part of root canals.

Luting resin thickness is significantly thinner in the anatomic posts with respect to standardized posts, even if in the apical third of the canal, there was no statistically significant difference. A thinner and more uniform luting resin layer is crucial. This was supported by Grandini et al., who reported that one of the major factors predisposing to void formation is the thickness of the endodontic luting resin, asserting that a greater thickness, especially in the coronal portion of the post, increases the tendency for post debonding. This implies that solid standardized posts necessitate better adaptation to the canal’s shape at the margins to achieve reduced luting resin thickness and therefore a lower chance of debonding [13,27].

In our sample, we noted in the working phases on the H group the need to use a important force to make the resin flow through the perforated body of the post; this is due to the viscosity of the luting resin itself, which must flow within a cylindrical canal of restricted diameter (approximately 0.5 mm). Despite this operational difficulty, the increase in pressure within the hollow portion of the post can be considered one of the factors capable of reducing air voids incorporated by the luting resin, which in this manner reaches the bottom of the preparation in a more homogeneous fluid and with a reduced number of empty spots [28].

The more the post occupies the space, the more retention it provides to the core [27]; hence, the elements from the hollow post group showed a better capacity to reach the most apical part of the preparation.

Although all the canals were straight and were prepared with the same techniques and tools, the hollow posts showed a greater propensity to occupy the post space entirely. Since there were no coronal interferences in any of the extracted teeth, the reason for this result could be derived from the different luting techniques: traditional luting of the fiber post is performed by first placing the luting resin into the canal, but by doing this, the layer of luting resin could create a sort of buffer layer, which limits its down-seating. In our study, the hollow post luting technique avoids this possibility, as luting resin is inserted in the post space existing through the inner cavity of the same post. This result suggests using hollow posts as an alternative approach to single fiberglass post techniques, even though further studies are necessary.

It should be noted that while self-etch luting resins are designed to simplify the bonding procedure by integrating etching and priming, pre-etching with phosphoric acid can often improve bond strength, especially in root canals or specific areas like the coronal third, or when dealing with the smear layer [29]. This is why the authors pre-treated the dentin with 30 s etching with 37% orthophosphoric acid.

## 5. Conclusions

Hollow posts have proved to be an easy-to-use and high-performance device in terms of their ability to go down into the post space. The reduced formation of air voids inside the luting resin and the ability to penetrate and fill the endodontic system are factors that contribute to retention and could contribute to good long-term results for post-endodontic reconstructions that require endocanalar retention. According to the results of this research, the disposal system of luting resin that uses the inner tube offered by hollow posts seems to be a key factor in reducing air voids.

Our results also confirmed that hollow posts led to a lower presence of voids inside the luting resin. A limitation of this study is that the sample size was not determined by a formal a priori calculation. However, the sample size of 10 specimens per group is consistent with similar in vitro dental studies. Furthermore, a post hoc power analysis demonstrated that our sample size was adequate to detect the observed differences with a statistical power of 80%, given the large effect size found for air void formation (Cohen’s d = 2.09). Nevertheless, future studies with larger samples are encouraged to confirm these findings and enhance their generalizability. Future studies with larger samples are encouraged to confirm these findings and enhance their generalizability.

Hollow posts seemed to perform better than solid posts, because of a lower presence of voids inside luting resin and between fiber post and luting resin itself. Nevertheless, in vivo long-term studies should also include chewing force, parafunctional events, the influence of occlusal contact with an opposing implant, different endodontic sealing materials, and exposure of the restored tooth to the oral environment [30,31,32,33,34] to enhance the comprehensiveness of the research.

## Figures and Tables

**Table 1:** SkyScan 1176 (Bruker™) CT specifications and exact parameters used in the void detection process.

**X-Ray Source**	**Sealed Microfocus X-Ray Tube 20–90 kV, 25 W**
X-ray detector	4000 × 2672 pixels, 12 bit, fiber optically coupled to scintillator
Scanning volume	68 mm diam., 20 mm single scan length, 200 mm scannable length
Spatial resolution	<9 µm pixel size, <15 µm low-contrast resolution (10% MTF)
Reconstructed slices	8 K × 8 K pixels, 9 µm/18 µm/35 µm selectable pixels size
Radiation safety	<1 µSv/h at any point on the instrument surface during scanning

**Table 2:** Different outcomes of empty spaces formation in the luting resin compared to total luting resin volume with the use of hollow posts and solid posts in shaped canals.

N°	Post Type	External Luting Resin Volume	Empty Luting Resin Space	% of Gaps	Empty Luting Resin Spaces/External Luting Resin Volume
1	HP	15.8300	0.5980	3.77%	0.0377
2	HP	10.0126	0.2741	2.27%	0.0227
3	HP	13.9505	1.4698	9.53%	0.0953
4	HP	7.3448	0.3655	4.73%	0.0473
5	HP	3.6510	0.1618	4.24%	0.0424
6	HP	3.5528	0.1150	3.13%	0.0313
7	HP	4.1995	0.0647	1.51%	0.0151
8	HP	3.5559	0.2975	7.72%	0.0772
9	HP	3.5768	0.2187	5.76%	0.0576
10	HP	4.4804	0.0317	0.07%	0.0070
11	SP	7.3413	1.4290	19.46%	0.1946
12	SP	7.0833	0.2775	3.91%	0.0391
13	SP	4.9525	1.3433	27.2%	0.2712
14	SP	6.2974	1.8191	28.8%	0.2888
15	SP	5.4689	1.1732	21.4%	0.2145
16	SP	6.3710	1.2366	19.41%	0.1941
17	SP	4.4895	0.8721	19.42%	0.1942
18	SP	6.2572	0.3396	5.43%	0.0543
19	SP	4.2698	0.8282	19.39%	0.1939
20	SP	11.3717	1.2342	10.85%	0.1085

**Table 3:** Different outcomes of penetration of hollow posts (HPs) and traditional solid posts (SPs) in shaped canals.

N°	Post Type	Post Length (mm)	Post Space Length (mm)	Post Length/Post Space Lenght
1	HP	7.595	7.729	0.9826
2	HP	8.157	8.22	0.9923
3	HP	6.318	7.148	0.8838
4	HP	8.693	9.059	0.9595
5	HP	7.747	8.247	0.9393
6	HP	7.702	8.372	0.9199
7	HP	8.344	8.478	0.9841
8	HP	8.264	8.577	0.9635
9	HP	8.059	8.416	0.9575
10	HP	7.881	7.979	0.9877
11	SP	6.212	8.157	0.7615
12	SP	6.961	8.541	0.8150
13	SP	5.551	8.594	0.6459
14	SP	4.658	7.639	0.6097
15	SP	4.373	8.273	0.5285
16	SP	5.971	8.39	0.7116
17	SP	6.696	7.069	0.9858
18	SP	7.033	8.246	0.8528
19	SP	7.417	7.783	0.9529
20	SP	4.736	8.362	0.5663

**Table 4:** Mann–Whitney test for Hp1 hypothesis.

Mann–Whitney Test	
0.0007	*p* value
Exact	Exact or approximate *p* value?
Yes	Significantly different (*p* < 0.05)?
Two-tailed	One- or two-tailed *p* value?
63, 147	Sum of ranks in column A,B
8	Mann–Whitney U

**Table 5:** Mann–Whitney test for Hp2 hypothesis.

Mann–Whitney Test	
0.0433	*p* value
Exact	Exact or approximate *p* value?
Yes	Significantly different (*p* < 0.05)?
Two-tailed	One- or two-tailed *p* value?
78, 132	Sum of ranks in column A,B
23	Mann–Whitney U

## Data Availability

The original contributions presented in this study are included in the article. Further inquiries can be directed to the corresponding author.
